# The knowledge and perceptions regarding antibiotic stewardship of the interns rotating at the Bloemfontein Academic Complex

**DOI:** 10.4102/safp.v63i1.5336

**Published:** 2021-10-26

**Authors:** Anke Archer, Marna Blom, Renette de Lange, Esther Jansen van Vuuren, Theunis E Kellerman, Samantha Potgieter, Gina Joubert

**Affiliations:** 1 Department of Family Medicine, Faculty of Health Sciences, University of the Free State, Bloemfontein, South Africa; 2 Division of Infectious Diseases, Department of Internal Medicine, Faculty of Health Sciences, University of the Free State, Bloemfontein, South Africa; 3 Department of Biostatistics, Faculty of Health Sciences, University of the Free State, Bloemfontein, South Africa

**Keywords:** antibiotic resistance, medical interns, knowledge, perception, education

## Abstract

**Background:**

Antibiotic resistance (ABR) is a global problem with the overuse of antibiotics accelerating this process. Antibiotic stewardship aims to optimise antibiotic treatment to enable cost-effective therapy and improve patients’ outcome whilst limiting ABR. The study aimed to evaluate intern medical doctors’ knowledge and perceptions about antibiotic stewardship and their perceptions regarding education on relevant topics.

**Methods:**

This was a cross-sectional study on interns rotating at Bloemfontein Academic Complex. An anonymous, self-administered questionnaire was completed. The questionnaire recorded demographic information, perception and knowledge of antibiotic stewardship, and the quality of education as perceived by the interns.

**Results:**

Of the 120 possible participants, 92 (76.7%) responded to all or part of the questionnaire. The median age of the respondents was 25 years, and 56.7% of the respondents were female. The mean score for the knowledge-based case scenarios was 5.4 out of 10. Only 4.4% participants could manage a drip site infection correctly, whilst 18.5% could treat *Escherichia coli* (*E. coli*) bacteraemia. The interns perceived that they have a lack of training and preparedness in certain areas of prescribing antibiotics. Though 77.2% of the interns had received education on starting antibiotic treatment, 29.3% claimed to be unsure when to start antibiotic therapy. Interns indicated that formal lectures (81.3%) and bedside tutorials (86.7%) have a high educational value.

**Conclusion:**

Intern medical doctors do not have sufficient knowledge to establish antibiotic stewardship but have a desire for improvement. The results identified specific areas where better antibiotic training is required.

## Introduction

Antibiotic resistance (ABR) is a worldwide problem with the overuse of antimicrobial substances accelerating this process.^1,2^ Prolongation of antibiotic treatment also promotes ABR, which can lead to extended infection periods, morbidity and mortality.^3,4^

The treatment of patients with resistant infections is more expensive than treatment and care for patients with non-resistant infections, with lengthier hospital stays and the need for more intensive care. In a healthcare system that is already struggling to cope with the burden of disease, the prolongation of hospital stay because of ABR will be devastating on a country’s healthcare system.^3,4^

Antibiotic stewardship can be defined as a set of strategies that encourage the practice of giving the correct antibiotics and dosage via the correct application route for the right amount of time.^2^ Antibiotic stewardship aims to optimise antibiotic treatment to enable cost-effective therapy and improve patients’ outcome whilst limiting ABR.^5^

The World Health Organization (WHO) has constructed an action plan regarding antimicrobial resistance that was presented at the May 2015 World Health Assembly. The action plan mainly consists of spreading awareness about ABR, improving knowledge through research, minimising infection by improving prevention strategies and increasing investment in interventions.^6^

Antibiotic stewardship education is crucial to provide foundational knowledge regarding antibiotics and ABR, making the correct diagnosis and infection management and appropriate prescribing. Education on appropriate prescribing for healthcare providers has shown to improve other antibiotic stewardship interventions.^7^ Improved communication skills will also help to convey this information to patients.^7^

In South Africa, the primary obstacle in antimicrobial stewardship programmes in almost all public and private health facilities has been insufficient resources and expertise about infectious diseases.^8^

Studies in Europe,^9^ Australia^10^ and South Africa^11^ have found that medical students have limited knowledge and confidence regarding antibiotic prescription. Similarly, studies in the United Kingdom^12,13^ have highlighted the needs and challenges faced by junior doctors (who frequently are the prescribers of antibiotics) regarding antibiotic prescribing.

This study aimed to determine the knowledge and perceptions of intern medical doctors in Bloemfontein regarding antibiotic prescribing and stewardship and their perceptions on the usefulness of different educational activities for learning about antibiotic-related topics.

## Materials and methods

### Study design, population and sampling

This was a cross-sectional study. The target population included all 120 first- and second-year intern medical doctors rotating at the Bloemfontein Academic Complex through eight departments from August 2017 to October 2017. The departments included: Family Medicine, Surgery, Anaesthesiology, Paediatrics, Orthopaedics, Obstetrics and Gynaecology, Internal Medicine, and Psychiatry.

### Data collection and measurements

An anonymous, self-administered questionnaire in English was handed out to interns at the end of a formal lecture after the study, and its aim was explained by the researchers. Lecturers assisted in questionnaire distribution and collection, ensuring that questionnaires were completed individually and not as group work. Completed questionnaires were handed in at the door.

Permission was granted by Dr Sean Wasserman (University of Cape Town) to use a questionnaire composed by researchers from the University of Cape Town who conducted a study on antibiotic stewardship on final-year medical students at three medical schools (University of the Witwatersrand, University of Cape Town and University of the Free State [UFS]).^11^ Some of the questions were altered to accommodate the difference between the study populations of the two studies.

The questionnaire consisted of sections on demographics (medical school attended, gender, age, previous qualifications), knowledge (self-reported awareness of antibiotic guidelines and the term antibiotic stewardship, 10 clinical case scenarios with five options to choose the correct answer from) and perceptions regarding antibiotics, their causes and outcomes, the usefulness of various educational activities to learn about antibiotic classes, antibiotic prescribing and resistance.

### Data analysis

Results were summarised by frequencies and percentages (categorical variables) and means, medians and interquartile ranges (IQRs) (numerical variables). The analysis was done by the Department of Biostatistics, Faculty of Health Sciences, UFS.

### Pilot study

A pilot study was conducted mainly to determine the amount of time needed to complete the questionnaire. The pilot study consisted of a group of 11 fifth-year medical students from UFS, who were selected on a single day as a convenient sample at National District Hospital. Questionnaires were handed out to the students after a formal lecture and were collected as the students left the venue. No changes were made to the questionnaire.

### Ethical considerations

The protocol was approved by the Health Sciences Research Ethics Committee, UFS (HSREC-S 13/2017). Permission was obtained from the Free State Department of Health to include intern medical doctors in this study.

## Results

Of the 120 interns, 92 (76.7%) responded to all or part of the questionnaire. The 28 interns who did not respond consisted of 11 who were on leave, 1 who refused and 16 who were unavailable during the time of data collection. The median age of the respondents was 25 years (IQR: 24–26 years) and 56.7% of the respondents were female. All South African medical schools were represented amongst the respondents with the UFS representing the largest percentage (34.1%). Five respondents indicated that they received their medical training outside South Africa.

### Knowledge

According to 59.3% (51 of 86) of the respondents, the hospitals where they work have antibiotic guidelines. The majority of respondents (81.5%, 75 of 92) indicated that they have used or consulted antibiotic guidelines.

Half (54.4%, 50 of 92) of the respondents reported being very familiar with the term ‘antibiotic stewardship’ and 34.8% reported being familiar, that is, they have heard the term mentioned in lectures or tutorials, but it was not explained further.

The mean score for the knowledge-based case scenarios was 5.4 out of 10. Only 6.5% of the respondents had a score of 8 or more out of 10 and 77.2% had a score of 5 or more out of 10.

Most of the respondents (76.1%, *n* = 70) knew what antibiotic to prescribe when managing an uncomplicated urinary tract infection. However, only 29.4% (*n* = 27) knew what antibiotic to prescribe for methicillin-sensitive *Staphylococcus aureus*.

The majority of the respondents (83.7%, *n* = 77) knew how to assess the severity and management of community-acquired pneumonia. Less than two-thirds (62.0%, *n* = 57) of the interns knew how to interpret an antibiogram and de-escalate antibiotic treatment, and the same percentage knew how to manage an upper respiratory tract infection.

The correct management of a drip site infection was only identified by 4.4% (*n* = 4) of the respondents. Half (47.8%, *n* = 44) knew how to correctly assess the severity and management of a *Clostridium difficile* infection.

The correct antimicrobial selection for extended-spectrum beta-lactamases (ESBL)-positive *Escherichia coli* bacteraemia was made by 18.5% (*n* = 17) of the respondents. Perioperative antibiotic prophylaxis duration and risks of excessive use were assessed correctly by 73.9% (*n* = 68) of the respondents. The majority of respondents (87.0%, *n* = 80) knew how to manage the poor response to empiric antibiotic therapy.

### Perceptions regarding antibiotic stewardship

Respondents’ answers regarding perceptions relating to antibiotic prescribing indicated that the majority were aware of ABR, its causes and outcomes and would like more education on ABR and appropriate use of antibiotics (Table 1). Only 50.0% agreed that lack of hand disinfection by healthcare workers causes the spread of ABR, and 29.4% chose the option neutral for this question. Although more than 50% disagreed with the statement that new antibiotics to deal with ABR are available, a quarter chose the option neutral.

**TABLE 1:** Summary of the respondents’ perceptions regarding antibiotic prescribing.

Statement	Disagree/strongly disagree		Neutral		Agree/strongly agree	
	*n*	%	*n*	%	*n*	%
Antibiotics are overused in South Africa	3	3.3	8	8.7	81	88.0
Antibiotics are overused at the hospitals where I have rotated	10	10.9	12	13.0	70	76.1
Antibiotic resistance is a significant problem in South Africa	3	3.3	10	10.9	79	85.9
Antibiotic resistance is a significant problem at the hospitals where I have rotated	5	5.4	29	31.5	58	63.0
New antibiotics are available to deal with the problem	54	58.7	24	26.1	14	15.2
Inappropriate use of antibiotics causes antibiotic resistance	0	0.0	2	2.2	90	97.8
Better use of antibiotics will reduce levels of antibiotic resistance	1	1.1	5	5.4	86	93.5
Use of broad-spectrum antibiotics when equally effective narrower spectrum antibiotics are available can increase antibiotic resistance	6	6.5	5	5.4	81	88.0
Lack of hand disinfection by healthcare workers causes the spread of antibiotic resistance	19	20.7	27	29.4	46	50.0
Inappropriate use of antibiotics is directly harmful to patients	3	3.3	15	16.3	74	80.4
A good knowledge of antibiotics is important to my work as doctor	0	0.0	1	1.1	91	98.9
I would like more education on antibiotic resistance	2	2.2	8	8.7	82	89.1
I would like more education on the appropriate use of antibiotics	1	1.1	10	10.9	81	88.1

### Perceptions regarding preparedness in antibiotic prescribing

As summarised in Table 2, most of the respondents reported that their medical school training prepared them well for making an accurate diagnosis (84.8%) and knowing when to start antibiotic treatment (70.7%). Only 17.6% felt that they were prepared on how to interpret antibiograms, whereas more than half (57.1%) rated their preparedness as poor or very poor. On most of these statements, between a quarter and a third of respondents chose the neutral option, with the highest percentage in this category being for the statement regarding knowledge of dosing and duration (42.4%).

**TABLE 2:** Interns’ self-reported perceptions regarding their preparedness to prescribe antibiotics (*n* = 92).

Statement	Good/very good		Neutral		Poor/very poor	
	*n*	%	*n*	%	*n*	%
Making accurate diagnosis	78	84.8	13	14.1	1	1.1
Knowing when to start antibiotics	65	70.7	25	27.2	2	2.2
Choosing the correct antibiotic	51	55.4	35	38.0	6	6.5
Knowledge of dosing and duration	40	43.5	39	42.4	13	14.1
How to de-escalate to narrower spectrum	35	38.0	33	35.9	24	26.1
How and when to transition from intravenous to oral	47	51.1	27	29.4	18	19.6
How to interpret antibiograms (*n* = 91)	16	17.6	23	25.3	52	57.1
Understanding spectrum of activity	48	52.2	28	30.4	16	17.4
Understanding basic mechanisms of resistance	55	59.8	30	32.6	7	7.6

### Perceptions regarding education on relevant topics

As indicated in Table 3, formal bedside tutorials were the educational method chosen by the largest percentage of respondents as being useful. More than 80% of the respondents also rated formal lectures, consultant ward rounds and problem-based learning as useful when it comes to antibiotic classes and spectrums of activity as well as antibiotic prescribing and resistance. Respondents found consultant ward rounds more useful than registrar ward rounds.

**TABLE 3:** Perceptions of the respondents regarding the usefulness of different educational methods in the medical curriculum.

Variable	Not useful at all/not useful		Neutral		Useful/very useful	
	*n*	%	*n*	%	*n*	%
**Antibiotic classes and spectrums of activity**						
Formal lectures (*n* = 91)	4	4.4	14	15.4	73	80.2
Formal bedside tutorials (*n* = 91)	3	3.3	8	8.8	80	87.9
Consultant ward rounds (*n* = 91)	5	5.0	10	11.0	76	83.5
Registrar ward rounds (*n* = 92)	12	13.0	18	19.6	62	67.4
Problem-based learning (*n* = 91)	6	6.6	9	9.9	76	83.5
**Antibiotic prescribing and resistance**						
Formal lectures (*n* = 91)	5	5.5	12	13.2	74	81.3
Formal bedside tutorials (*n* = 90)	2	2.2	10	11.1	78	86.7
Consultant ward rounds (*n* = 91)	5	5.5	10	11.0	76	83.5
Registrar ward rounds (*n* = 92)	12	13.0	20	21.7	60	65.2
Problem-based learning (*n* = 90)	6	6.7	12	13.3	72	80.0

The majority of respondents had attended lectures or tutorials for the following: diagnosis of infection (85.9%, *n* = 79), indications for starting antibiotics (77.2%, *n* = 71), dosing and duration of antibiotic therapy for specific infection (76.1%, *n* = 70), indications for intravenous antibiotics (78.3%, *n* = 72), rational use of antibiotics in general (78.3%, *n* = 72) and basic principles of infection prevention and control (85.9%, *n* = 79).

The respondents indicated that a local handbook or guidelines on treating common infections (78.3%, *n* = 72), more applications for smart devices (58.7%, *n* = 54) and more exposure to infectious diseases specialists (53.3%, *n* = 49) were ways to prepare newly qualified doctors to prescribe antibiotics. Less than half suggested more formal bedside tutorials (41.3%, *n* = 38), more formal lectures (31.5%, *n* = 29) and more time in microbiology (25.0%, *n* = 23). Only 10.9% (*n* = 10) selected ‘more computer-based tutorials’ as a preparation method.

## Discussion

Of the 120 possible candidates, 76.7% responded to all or part of the questionnaire. This is considered a good response rate and was in line with the 80% reported for UFS and the 75% reported for the University of Cape Town in the study conducted on the final-year medical students.^11^

### Knowledge

In the study done on final-year medical students,^11^ which used the same clinical case scenarios, the mean correct score was 50.0%. There is thus little difference in knowledge between final-year medical students and intern medical doctors (5.4 out of 10). It can be assumed that most of the interns’ knowledge is acquired during training in medical school.

From the knowledge questions, it was clear that interns knew what antibiotics to prescribe for a urinary tract infection and how to assess the severity and management of community-acquired pneumonia. This is in accordance with the South African final-year medical student study.^11^ This can be expected as urinary tract infections and pneumonia are relatively common diseases in South Africa.^14^

In a clinical case scenario of the correct antibiotic use for methicillin-sensitive *Staphylococcus aureus*, only 29.4% of interns knew what antibiotics to prescribe. A reason for this low score could be the fact that the question was not read carefully. The term ‘methicillin-resistant’ is usually used when referring to ABR. Thus, the term ‘methicillin-sensitive’ may have been misread. It should be noted, however, that this correlates with the fact that only 54.4% of respondents felt adequately prepared to prescribe antibiotics.

In the clinical case scenario, the correct management of upper respiratory tract infection was reported by 62.0% of interns. This percentage is lower than expected because upper respiratory tract infections are commonly seen,^15^ and it would be expected that most interns would know how to manage it correctly. Most upper respiratory tract infections are caused by viral infections where antibiotic prescribing will have no effect. Research has demonstrated high rates of unnecessary antibiotic prescribing for these types of infections, which promotes ABR.^15^ In order to promote antibiotic stewardship, better management of upper respiratory tract infections is needed.

The correct management of a drip site infection was only identified by 4.4% of the interns. Serious intervention is needed to improve the management of drip site infections as it is a fairly common occurrence in hospital environments.^16^

Less than half of the respondents (47.8%) knew how to correctly assess the severity and management of *Clostridium difficile* infection. Better management of the infection is needed as *Clostridium difficile* is the number one causative agent of antibiotic-associated and healthcare-associated infective diarrhoea in hospital environments.^16^

The correct antimicrobial selection for ESBL-positive *Escherichia coli* bacteraemia was made by 18.5% of interns. As extended spectrum infections are becoming more common as a result of ABR,^11^ correct treatment is needed to contain ABR. Overall, knowledge about antibiotic prescribing regimens and ABR are moderate to poor, which correlates with the South African final-year medical student study.^11^

The responses to the clinical case scenarios in the study questionnaire showed that some interns struggle to choose the right antibiotic treatment although 55.4% indicated that they felt prepared to choose the correct antibiotic.

### Perceptions

The majority of intern medical doctors (85.9%) in this study realised that ABR is a problem in South Africa, but only 63.0% perceived ABR as a problem in hospitals where they have rotated. Studies have shown that a large number of medical students or doctors are ignorant to the fact that ABR is a problem in their immediate environment and this may contribute to over-prescription.^11,17^

Nearly a fifth of the respondents (19.6%) disagreed that the unnecessary use of broad-spectrum antibiotics increases ABR. As one of the main factors of increased ABR is the unnecessary use of antibiotics, interns need to become more aware about the causes of ABR. It is encouraging to see that the majority (89.1%) of interns indicated that they would like more education on ABR.

In our study, interns indicated that they lack training and preparedness in certain areas of antibiotic prescribing. This finding is in accordance with the study done on final-year medical students in South Africa.^11^ In a study done on junior doctors in London,^12^ less than 15% of participants indicated that their previous antimicrobial education was effective, and 60% of participants who had qualified the previous year stated that they required further education in antimicrobial prescribing. This latter percentage was 74.0% for more experienced junior doctors. Specific topics identified for further education in that study were: ‘(i) principles of antimicrobial prescribing, (ii) diagnosis of infections, (iii) clinical review of patients with infections, (iv) prescribing in the context of antimicrobial resistance and (v) laboratory testing and test results’ (p. 9).^12^

Three quarters of participating interns attended lectures or tutorials on the dosing duration of antibiotic treatment for specific infections. However, less than half of interns felt like they were prepared when it comes to the dosing duration of antibiotics. This gap can possibly be attributed to ineffective educational methods, too little time spent on preparing interns for dosing duration or intern medical doctors not frequently educated on the subject throughout their undergraduate training and career.

Half of the interns felt very prepared on how and when to transition from intravenous to oral antibiotic treatment compared to nearly 80% of the interns who reported to have had education on the indications of intravenous antibiotics. The disparity between education and knowledge is once again apparent.

Only 59.8% of interns have a perceived high understanding of the basic mechanisms of ABR; a fact that is troubling because understanding what causes resistance is an important way to curb ABR. When interns do not know that some of their actions can promote ABR, they will continue to unintentionally contribute to the problem. The fact that only 50.0% interns agreed that poor hand hygiene to ABR is concerning. This correlates with the final-year medical student study.^11^

### Education

Interns’ responses to the various available education’s effectiveness give an indication of what they would prefer. The majority reported that formal lectures, formal bedside tutorials, and consultant ward rounds are useful for learning about antibiotic spectrums, resistance and prescribing. Areas where intern medical doctors felt the least prepared were an interpretation of antibiograms, knowledge of dosing duration and performing de-escalation in antibiotic therapy. In the study done on final-year medical students, these were also the areas in which students felt the least prepared.^11^

Interns’ suggestions regarding useful approaches to prepare them for prescribing antibiotics place emphasis on alternative approaches rather than formal classes. An Australian study on junior doctors found that an educational session in conjunction with pocket guidelines improved self-confidence regarding vancomycin prescribing and monitoring.^18^

### Study strengths and limitations

The study had a high response rate and made use of an existing questionnaire that enabled direct comparison with the recent South African study on final-year medical students. Whilst evaluating the theoretical knowledge of antibiotic dosing and duration, it should be acknowledged that interns in the clinical setting make use of resource material such as the Internet to guide them. This could indicate that they would fare better in practice than shown in this study.

### Recommendations

For antibiotic stewardship programmes to improve, attention must be given to each aspect of antibiotic stewardship. More attention must be given to choosing the correct antibiotics and dosage, via the correct application route for the right amount of time, as well as better infection control. Better inpatient care practices should be taught and applied so that hand hygiene is not one of the leading causes of ABR.

There is a definite gap between undergraduate training, theoretical knowledge and clinical knowledge in intern medical doctors. Certain areas of medical value should be emphasised more. Changes need to be made in the educational method in which interns learn about starting antibiotic treatment and the dosing duration of antibiotic therapy. Ward rounds can focus more on the indications for intravenous antibiotic treatment and when to transfer to other application routes.

Intern medical doctors should be made more aware of the basic mechanisms of ABR, as this study has proven that most interns admit to not knowing the fundamental principles of ABR. This can be achieved through training during weekly meetings or training during medical school.

All doctors should be made aware, preferably at an early stage during medical school training, that ABR is a problem in their immediate environment to promote the applicability of antibiotic stewardship programmes. Small steps can be taken to improve antibiotic stewardship programmes such as promoting the practice of proper hand hygiene routines.

Educational methods in the medical curriculum should be adapted according to indications of what interns and final-year medical students would prefer.

Future research should investigate factors contributing to poor antibiotic prescribing knowledge amongst intern medical doctors.

## Conclusion

Our results show that intern medical doctors do not have basic antibiotic prescribing knowledge, but there is a desire for more education in this area. Provision of more information and adapted teaching methods for interns may contribute to a reduction in the prevalence of ABR and promote antibiotic stewardship.
